# Paget-Schroetter Syndrome: A Case of a Young Weightlifter

**DOI:** 10.7759/cureus.62824

**Published:** 2024-06-21

**Authors:** David T Crossland, Matthew D Overturf

**Affiliations:** 1 Medicine, Edward Via College of Osteopathic Medicine, Monroe, USA; 2 Anatomical Sciences, Edward Via College of Osteopathic Medicine, Monroe, USA

**Keywords:** catheter-directed thrombolysis, thoracic outlet syndrome, vascular surgery, upper extremity deep venous thrombosis, paget-schroetter syndrome

## Abstract

Venous thoracic outlet syndrome is a rare type of thoracic outlet disorder that is often overlooked. When an upper extremity deep vein thrombosis (UEDVT) occurs due to thoracic outlet compression, it is commonly referred to as Paget-Schroetter syndrome (PSS). The space between the first rib and the clavicle where the subclavian vein passes through is highly vulnerable to compression and injury. This space often undergoes repetitive trauma due to extrinsic compression which ultimately results in scarring and clot formation. This case report reviews the case of a 26-year-old white male who presented with the chief complaint of right arm swelling and soreness after strenuous bench pressing. He went to urgent care and the initial diagnosis was a strained muscle. An ultrasound was ordered, revealing multiple UEDVTs. At this time, the patient was referred to vascular surgery for further management. Recommended management for PSS is to initiate anticoagulation or thrombolytic therapy depending on the timing between the onset of symptoms and diagnosis. Although there is some disagreement on the next steps after thrombolysis, most physicians agree that decompression of the thoracic outlet with first rib resection is the logical next step to prevent clot recurrence. The patient received the above-mentioned therapy and is progressing well with recovery. Recognizing the thoracic outlet as a potential location for pathology and keeping in mind those who have a presentation similar to this case study is extremely important.

## Introduction

Upper extremity deep vein thrombosis (UEDVT) is divided into two categories: primary and secondary. Primary UEDVT includes Paget-Schroetter syndrome (PSS) and idiopathic thrombosis, whereas secondary UEDVT refers to thrombosis caused by congestive heart failure, malignant disease, drug abusers, or patients with pacemakers or venous catheters [1].

PSS is uncommon, with an incidence of approximately 0.5-1 per 100,000 people annually [1]. It most commonly occurs in patients who perform strenuous overhead activities, such as weight trainers, swimmers, baseball pitchers, and auto part mechanics [1]. PSS manifests due to the possibility of compression of the subclavian vein as it passes between the first rib and the clavicle [1]. Increased muscle hypertrophy or abnormal osseous arrangement increases the risk for vein compression, and, subsequently, clot formation [1]. PSS does not threaten the limb and has a low probability of pulmonary embolism, but it can cause serious disability, such as arm swelling, pain, and early exercise fatigue, in the long term if not treated [2].

The most common first step in UEDVT is anticoagulation therapy with either low-molecular-weight heparin or oral anticoagulants such as apixaban or rivaroxaban [3]. Catheter-directed thrombolysis is also an alternative first line of treatment for thoracic outlet syndrome patients who present with moderate-to-severe symptoms, especially if the thrombus is detected within two weeks of the onset of symptoms [4-6]. Surgical decompression of the thoracic outlet via first rib resection following thrombolysis is thought to be an effective treatment option resulting in excellent patency rates [4-7].

This is a case report addressing a singular patient’s presentation while considering different evidence-based therapy options. In doing so, the report aims to increase physician awareness with the hope of improving the treatment of future patients with similar presentations.

## Case presentation

On December 7, 2023, a 26-year-old white male presented to a primary care clinic with the chief complaint of right arm pain and swelling after exercising the day before. He stated it felt like he had a tennis ball in his axilla. He reported that he was doing strenuous bench pressing, completed his workout, and then woke up the next morning with a discolored, tender right arm. The patient denied any pertinent past medical history or surgical history. An upper extremity ultrasound was to be scheduled for a later date and the patient was released.

On December 9, 2023, the patient went to a local urgent care due to persistent and increasingly severe pain and swelling. An upper extremity X-ray revealed no acute osseous abnormalities. As the assumption was that the patient had developed a tendinopathy, he was prescribed oral corticosteroids and released.

**Figure 1:**
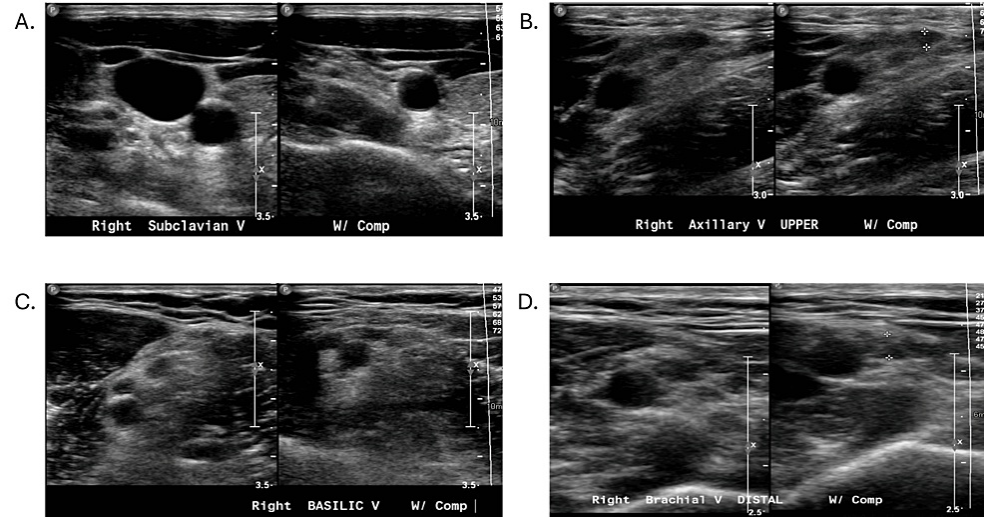
Duplex ultrasound of the right upper extremity reveals multiple acute deep vein thromboses. Duplex ultrasound of the right upper extremity reveals a thrombosis in the subclavian vein (A), axillary vein (B), basilic vein (C), and brachial vein (D). Thrombosis is indicated by the lack of collapse of each vein upon compression, as noted on the right side of each sonogram.

On December 18, 2023, a duplex ultrasound of the right upper extremity revealed multiple acute deep vein thromboses in the right subclavian vein, axillary vein, basilic vein, and one of the brachial veins (Figure 1). It was at this point that the primary care physician contacted a local vascular surgeon to assist in diagnostic management. The patient was prescribed 10 mg apixaban twice daily for seven days followed by 5 mg apixaban twice daily and referred to vascular surgery.

On December 21, 2023, the patient met with a vascular surgeon in the outpatient setting. Due to the longevity of the thrombus, immediate admission was recommended for thrombectomy with subsequent thoracic outlet decompression versus simple anticoagulation. After discussing the long-term possible symptoms if left untreated, the patient opted for both thrombectomy and surgery. At this point, the thrombus was two weeks old, which was the reason for the same-day admission.

Upon admission, multiple labs were drawn to assess for an inherent cause of hypercoagulability. These included factor V Leiden, factor II (prothrombin G20210A), anticardiolipin antibodies, anti-β2 glycoprotein I antibodies, and lupus anticoagulant. Thrombophilia lab results were negative. He was started on intravenous heparin. The following day he was transported to the operating room (OR) for a right upper extremity venogram and thrombolytic therapy.

Venography with intravenous sheath placement was completed first. The patient was then given alteplase 10 mg continuous intravenous infusion at a rate of 1 mg/hour to aid in clot breakdown and was admitted to the intensive care unit (ICU). The patient was transported to the OR the next day for sheath removal. Upon removal of the sheath, residual stenosis was noted in the subclavian vein, consequently, balloon angioplasty was performed. Repeat contrast venography demonstrated improvement. The patient was discharged on December 24, 2023.

On December 27, 2023, the patient returned to the hospital for first rib resection and thoracic outlet decompression. A surgical drain was left in place for one day to allow fluid to drain and decrease the risk of infection. Overall, the procedure was successful, and the patient was discharged.

The plan is for the patient to follow up with the vascular surgeon in two weeks and then again after three months when another upper extremity ultrasound will be done to reassess venous patency. He will remain on 10 mg apixaban daily for three months.

## Discussion

Epidemiology

Deep vein thrombosis (DVT) is a common condition with an incidence of 48-124 cases per 100,000 people per year [8]. DVT of the upper extremity, however, is a rare disorder and is estimated to range from 1% to 4% of all cases of DVT [1]. The incidence has increased in the past few decades due to the use of central venous catheters and cardiac pacemaker placement [1]. PSS is a type of primary UEDVT that is the result of compression most commonly of the subclavian or axillary vein, which causes obstruction and thrombosis [9]. Primary UEDVT is a rare condition; the incidence is estimated to be between 1 and 2 for every 100,000 patients per year with a predilection for males over females at a rate of 2:1 [9]. Because primary UEDVT is often associated with physical activity and congenital anatomic abnormalities, the median age of diagnosis tends to be on the younger side at approximately 30 years [9]. Secondary UEDVT occurs more commonly in the subclavian vein but also occurs in the jugular, axillary, brachial, or brachiocephalic veins [9]. Secondary UEDVT is caused mostly by malignancy or catheter placement. The most important risk factors are malignancy (prevalence 24-65%) and indwelling lines (10-93%) [9]. Additional risk factors are recent surgery, trauma, use of hormone therapy, and hereditary or acquired thrombophilia [9]. Because the two biggest risk factors for secondary UEDVTs are cancer or central venous catheters, the median age of diagnosis is 60 [9]. One of the largest studies done on UEDVTs, the MATS study, examined 1,203 patients with venous thromboembolism from March 1998 to December 2006, and 63 (5%) of these patients had UEDVT [8]. Research indicates that UEDVT is a rare diagnosis, and PSS is even rarer. The diagnosis of UEDVT was confirmed with phlebography, duplex ultrasound, CT, or MRI [8]. Of the 63 patients diagnosed with UEDVT, 33 (52%) were classified as primary, with only one patient being classified as PSS and the other 32 classified as idiopathic [8]. Thirty (48%) patients were classified as having secondary UEDVT [8]. Of the secondary UEDVT cases, 17% were iatrogenic, 21% had malignancy only, and 9% had both malignancy and catheter placement [8].

Anatomy and pathophysiology

**Figure 2:**
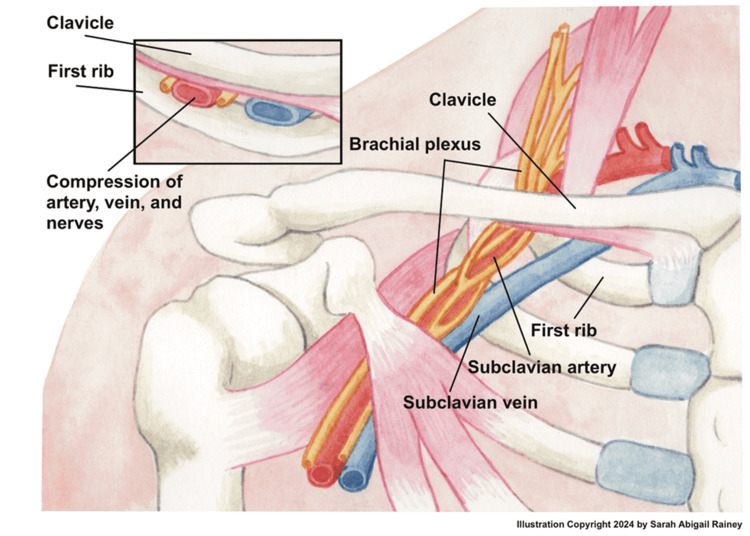
Basic anatomy of the thoracic outlet where Paget-Schroetter syndrome occurs. As you move distal to proximal, the axillary vein becomes the subclavian vein which can be compressed superiorly by the clavicle and inferiorly by the first rib. The subclavius muscle can be a site for compression medially and laterally the anterior scalene muscle. Illustration reproduced with permission. Copyright 2024 Sarah Abigail Rainey.

PSS occurs in the anterior part of the thoracic outlet region, where the subclavian vein passes by the intersection of the clavicle and the first rib (Figure 2). The posterior portion of this area is home to the anterior scalene muscle, which can compress the subclavian vein from behind, while the subclavius muscle, which sits right below the clavicle, can compress the outlet from down below [4]. It is unclear which of these structures is the primary cause of compression. Adams et al. revealed that the subclavian vein can be compressed with just abduction of the arm [10]. Because of this, it can be a challenge to understand where the true location of pathology lies.

Young healthy patients with PSS most commonly develop spontaneous UEDVT in the dominant arm [11]. This usually occurs after strenuous activities that require recurrent abduction of the arm, such as baseball pitching, basketball shooting, weightlifting, or rowing [11]. The suggested theory is that the heavy exertion causes microtrauma to the vascular intima which leads to activation of the coagulation cascade [11]. If the insults are repeated and there is significant mechanical obstruction, thrombosis is likely to occur [11].

The most widely held theory regarding the cause of PSS is that patients have intermittent venous outflow obstruction caused by anatomic compression to the subclavian vein [9]. These patients may not have any evidence of actual injury to the vein [9]. However, recurrent and chronic compression can cause inflammation and then fibrosis in the structures around the vein [9]. This can lead to increased external compression on the vein which decreases the vein’s mobility. The progression toward thrombosis occurs with repetitive trauma and increased compression. Another theory is that thrombosis is the result of a congenital anatomic compression with a single inciting event [9]. Either way, both compression and inflammation play a role in the formation of clots.

Clinical presentation

The patient discussed was completely asymptomatic until they developed thrombosis. In other patients, there can be symptoms of intermittent venous thoracic outlet compression such as episodic arm discoloration and swelling [4]. These symptoms are usually elicited by strenuous exercise or arm abduction [4]. Gender predilection for PSS is debated, and the right arm more commonly develops thrombosis than the left [4].

There is often an inciting activity that results in the patient complaining of a swollen and heavy arm [4]. The arm may be blue or red and may change in color from red to blue as the thrombosis remains [4]. There also may be collateral superficial veins that are visible and prominent on the shoulder and arm going down [4]. According to one study, 60-80% reported a history of vigorous activity before the inciting incident and approximately 85% of patients had symptoms within 24 hours [4].

Diagnostics

When diagnosing PSS, a screening tool often utilized is a D-dimer test. One study demonstrated that abnormal D-dimer levels have a sensitivity of 92% and a specificity of 60% for UEDVT [9]. In patients with low clinical suspicion for UEDVT, a negative D-dimer test is extremely helpful in ruling out UEDVT. However, in patients where there is high clinical suspicion for UEDVT, additional imaging is recommended to rule it out.

Ultrasound and venography are the most common methods used to diagnose PSS, with the most effective being venography [9]. Though venography is considered the more effective method of diagnosis, it is invasive, is not always available, and has an increased risk of thrombophlebitis [9]. The most frequently used diagnostic tool is ultrasonography, but its reliability is debated [12]. The effectiveness of ultrasonography depends heavily on the technician and their anatomical knowledge, as well as how accessible the vein is along its course up the arm [12]. One study determined that ultrasound sensitivity for UEDVT ranged from 56% to 100% and specificity from 77% to 100% [12]. Because of this, clinical suspicion is important when approaching UEDVT.

One diagnostic tool that can be utilized is a diagnostic algorithm for UEDVT supported by the ARMOUR study [9]. In this algorithm, clinical suspicion of UEDVT in high-risk patients automatically calls for compression ultrasonography [9]. A patient not at high risk for UEDVT either is referred for D-dimer testing or compression ultrasonography, depending on their Constans score, a clinical probability score similar to the Wells criteria [9]. This algorithm has been shown to be useful in diagnosing patients with UEDVT.

Management

Summary

There are four methods for treating PSS. The first is anticoagulation therapy only. However, there is evidence that thrombolysis is far more effective in the long term than anticoagulation [4]. PSS treated with only anticoagulation causes residual upper extremity venous obstruction in 78% of cases and persistent symptoms with permanent disability are common [4]. One study showed that only 29% of patients treated with anticoagulation alone had a good long-term result [4]. Because of this, this case study will discuss the three methods that utilize thrombolysis, which uses a catheter-directed thrombolytic agent to break down the thrombi.

Option 1: Thrombolysis With Anticoagulation Without Surgical Decompression

PSS can be treated with thrombolysis and anticoagulation only. There are several arguments against this option. It does not treat the extrinsic compression of the vein and leaves the patient with the likelihood of developing thrombosis again. The long-term prognosis of UEDVT treated with only anticoagulation is poor. Side effects range from pulmonary emboli to chronic pain, swelling, and easy fatiguability of the affected extremity [4]. Still, one study indicated that this method can be effective in treating PSS. Nine patients who underwent thrombolysis followed by anticoagulation only were compared with 13 patients who underwent thrombolysis, had surgical intervention, and then had anticoagulation for one month [13]. After two years, eight of the nine patients treated with thrombolysis and anticoagulation alone reported minimal symptoms, while one reported moderate symptoms [13]. Of the 13 who had surgical intervention, six reported moderate symptoms, while two reported severe symptoms [13]. Neither group had thrombotic reoccurrence [13]. This study also noted five postoperative complications in the group that underwent surgery, including two cases of pneumothoraxes, one case of chylothorax requiring thoracentesis, and two cases of asymptomatic transient phrenic nerve palsy [13]. Anticoagulation has been reported as effective and is without risk of postoperative complications.

Option 2: Thrombolysis and Placement of Intravenous Stents

PSS can also be treated by thrombolysis followed by placement of stents. This method is flawed in that it has been shown to have a high probability of re-obstruction or re-fibrosing [4]. It is essential to remember that the force of the costoclavicular junction can easily compress and overcome the strongest stent. One study conducted in 2002 examined 22 patients who had intravenous stents inserted at outside hospitals [14]. These 22 patients were compared with a similar group of 384 patients who were treated with thrombolysis and first rib resection [14]. Within six weeks, all 22 patients treated with intravenous stents formed re-occlusions and had to undergo thrombolytic therapy with first rib resection [14]. Their long-term outcomes were not good, as five of the 22 patients are now on lifelong anticoagulation, in contrast to the 384 patients managed with surgery who did not need recurrent treatment or prolonged use of anticoagulants [14].

Option 3: Optimal Therapy - Thrombolysis With Decompression by First Rib Resection

The third option, and arguably the most effective option, for treating PSS is thrombolysis followed by surgical decompression and vein patching, with a short course of anticoagulation. Arguments against surgical intervention highlight postoperative risk, as discussed in Option 1. In addition to this, success is limited if the thrombus has been present longer than two weeks [4]. Catheter-directed thrombolysis is successful in 62-84% of patients and the rate is much higher the fresher the clot [4]. When looking at delayed treatment, one study at the University of Rochester found that no patient with symptoms longer than 10 days had successful thrombolysis [15]. Another study at Baylor showed 50% success if treated more than six weeks after symptom onset [16]. There is also evidence pointing to the wide-range success of this method. A meta-analysis of 12 case series was done on a total of 684 patients who underwent treatment for PSS [9]. It found that 95% of patients treated with decompression had symptom relief, compared to 54% of those with thrombolysis alone [9]. They also found that there was vein patency maintained at follow-up in 98% of the thrombolysis and decompression group compared to 48% in the thrombolysis and anticoagulation group [9].

Timing of decompression: The timing of rib resection following thrombolysis has been the subject of controversy in recent years. One recent systematic review looked at six different case series that had a total of 126 patients [9]. The meta-analysis divided the patients into two groups: early surgical intervention (less than two weeks after thrombolysis) and late surgical intervention (more than two weeks after thrombolysis). At the end of follow-up, 89% of patients in the early intervention group had minimal or no symptoms compared to 90% in the postponed intervention group. During follow-up, 18% of the early group had a recurrent event, while 31% of the postponed group had a recurrent event [9]. Based on this, the authors of the meta-analysis concluded that early intervention was possibly preferable to postponed intervention, but that either option could be defended.

Postoperative management and evaluation: Most physicians agree that these patients need to be anticoagulated [4]. Because the extrinsic compression should be removed and thus the cause of clots, short-term anticoagulation seems to be an acceptable choice [4]. Some studies recommend three months of anticoagulation, and some recommend six months, but most fall within the three- to six-month window [4]. Protocol also recommends an ultrasound at one to six months post-surgery with annual follow-up appointments to reassess the clinical presentation [4]. Long-term outcomes for surgical intervention appear successful. One study that followed patients for over three years found that surgical intervention resulted in successful treatment outcomes in over >90% of patients [17]. While clot reoccurrence can occur, success is the more common outcome.

## Conclusions

When approaching patients who are young and active and present with swelling and discoloration of their arm after strenuous activity, it is important to keep in mind the possibility of PSS. When looking at this case study, there was a significant delay between when the thrombosis occurred and when treatment was initiated. With PSS, it is important to remember that time is a significant factor. When diagnosing PSS, ultrasound is the first diagnostic step for those at high risk, but the results rely heavily on technician acumen. Because of this, one should remain skeptical of the results and clinical suspicion should drive management. D-dimer screening is useful for those who are at low risk of PSS, but still have a similar clinical presentation. Moreover, the most effective method of diagnosis is venography. Concerning treatment of PSS, both thrombolysis followed by anticoagulation and thrombolysis with decompression have been shown to be successful. However, evidence points to catheter-directed thrombolysis and first rib resection as the optimal treatment plan. As discussed before, the patient reviewed in this case study underwent optimal therapy and tolerated the procedures well. Though the clots had been present for just over two weeks, thrombolysis was successful. Moving forward, management of PSS should acknowledge the importance of swift diagnosis and doctors should be considerate of which treatment options have been more successful in the past.
